# Bacterial Nanocellulose Functionalized with Graphite and Niobium Pentoxide: Limited Antimicrobial Effects and Preserved Cytocompatibility

**DOI:** 10.3390/membranes16010016

**Published:** 2025-12-31

**Authors:** Juliana Silva Ribeiro de Andrade, Adriana Poli Castilho Dugaich, Andressa da Silva Barboza, Maurício Malheiros Badaró, Pedro Henrique Santaliestra e Silva, Tiago Moreira Bastos Campos, Karina Cesca, Debora de Oliveira, Sheila Cristina Stolf, Rafael Guerra Lund

**Affiliations:** 1 Department of Dentistry, Federal University of Santa Catarina (UFSC), Florianópolis 88040-535, SC, Brazil; adrianapoli@gmail.com (A.P.C.D.); andressahb@hotmail.com (A.d.S.B.); mauricio.badaro@ufsc.br (M.M.B.); karinacesca@gmail.com (K.C.); debora.oliveira@ufsc.br (D.d.O.); stolfsheila@gmail.com (S.C.S.); 2 Department of Restorative Dentistry, School of Dentistry, Federal University of Pelotas (UFPel), Pelotas 96015-560, RS, Brazil; pedrohssufpel@gmail.com (P.H.S.e.S.); moreiratiago22@gmail.com (T.M.B.C.)

**Keywords:** bacterial nanocellulose, chronic wounds, biomaterials, functionalized graphite, niobium pentoxide, bioactive composites, antimicrobial, cytocompatibility, spectroscopic characterization

## Abstract

Chronic wounds remain locked in persistent inflammation with high microbial burden, demanding dressings that suppress infection without sacrificing biocompatibility. Bacterial nanocellulose (BNC) is an attractive matrix due to its biocompatibility, nanofibrillar architecture, and moisture retention, but it lacks antimicrobial activity. Here, we engineer BNC membranes post-functionalized with functionalized graphite (f-Gr; predominantly graphitic with residual surface groups) and/or niobium pentoxide (Nb_2_O_5_), and evaluate four groups: BNC (matrix control), BNC/Nb_2_O_5_, BNC/f-Gr, and BNC/f-Gr/Nb_2_O_5_. Physicochemical analyses (Raman and Voigt fitting, FTIR-ATR, XRD, and SEM) confirm a graphitic carbon phase and physical incorporation of the modifiers into the BNC network, with a noticeable shift in the hydration/polarity profile—more evident in the presence of f-Gr. In standardized microbiological assays, BNC/f-Gr promoted a moderate, contact-dependent reduction in bacterial proliferation, particularly against *Staphylococcus aureus*, whereas BNC/Nb_2_O_5_ behaved similarly to pristine BNC under the tested conditions. The combined f-Gr/Nb_2_O_5_ formulation showed an intermediate antimicrobial response, with no clear synergy beyond f-Gr alone. Cytotoxicity assays indicated cytocompatibility for BNC, BNC/f-Gr, and BNC/Nb_2_O_5_; the combined group displayed a slight reduction that remained within acceptable limits. Overall, BNC/f-Gr emerges as the most promising antimicrobial dressing, while Nb_2_O_5_ did not significantly enhance antimicrobial performance under the tested conditions and warrants further optimization regarding loading and distribution.

## 1. Introduction

Chronic wounds represent a significant clinical and socioeconomic challenge, affecting millions of individuals globally. Characterized by persistent inflammation, high microbial burden, and compromised healing, these lesions lead to severe complications such as recurrent infections and the risk of amputation, drastically impacting quality of life and generating substantial costs for healthcare systems [1,2]. In this context, the development of functional membranes for advanced wound dressings is crucial, requiring materials that optimize infection control, promote tissue compatibility, maintain an ideal moist microenvironment, and provide structural support for tissue regeneration.

Bacterial nanocellulose (BNC) stands out as a versatile and promising platform for the design of biomedical membranes. Its intrinsic biocompatibility, nanofibrillar structure mimicking the extracellular matrix (ECM), exceptional water retention capacity, controllable porosity, and remarkable mechanical stability in the hydrated state qualify it as a membrane matrix of choice [3,4]. However, pristine BNC lacks certain essential functionalities for complex chronic wound management, notably robust intrinsic antimicrobial activity and, in specific scenarios, electrical conductivity or enhanced mechanical strength. This gap drives the investigation of its functionalization with strategic additives.

Carbonaceous materials have been extensively explored as membrane modifiers due to their ability to confer conductivity, modulate surface properties, and influence cellular and microbial interactions. Particularly, their application in scaffolds suggests that electrical and surface properties can optimize the repair process and infection control, even under electrical stimulation [5,6]. In this work, we employed functionalized graphite (f-Gr), a predominantly graphitic material with residual functional groups. This deliberate choice, distinct from graphene or graphene oxide, is informed by existing literature and aims to confer upon the BNC membrane the ability to modulate polarity and hydration, introduce conductive pathways, and influence bacteria–surface interactions, thereby contributing to microbial control without compromising the cytocompatibility of the composite membrane.

Concurrently, niobium pentoxide (Nb_2_O_5_) has been reported as an inorganic additive capable of modulating surface properties, bioactivity, and mechanical stability in different biomaterial systems, rather than acting as a primary antimicrobial agent. Its antibacterial performance has been shown to be highly dependent on composition, particle dispersion, exposure conditions, and the surrounding matrix. In polymer-based and ceramic composites, Nb_2_O_5_ has been associated with changes in surface energy, protein adsorption, and cell–material interactions, which may indirectly influence microbial adhesion. Based on this background, Nb_2_O_5_ was incorporated in the present study as a surface and structural modulator, and not as a standalone antimicrobial phase.

In this work, we employed functionalized graphite (f-Gr), a predominantly graphitic material with residual surface functional groups. Unlike graphene oxide, which exhibits a high oxidation degree and has been associated with dose-dependent cytotoxicity, f-Gr offers greater structural stability and a lower density of oxygenated defects. This choice was intentional to promote surface and hydration modulation while minimizing potential cytotoxic effects, making f-Gr more suitable for preliminary screening in contact-based antimicrobial membranes. To this end, four formulations will be compared: BNC (matrix control), BNC/Nb_2_O_5_, BNC/f-Gr, and BNC/f-Gr/Nb_2_O_5_. The central hypothesis is that the post-synthesis incorporation of f-Gr and Nb_2_O_5_ will modify the surface properties of BNC, reducing bacterial proliferation compared to the pristine matrix. This study serves as a preliminary investigation into the physicochemical interactions and biological safety of these composites for potential use in wound management.

## 2. Materials and Methods

### 2.1. Materials and Chemicals

Functionalized graphite (f-Gr) was kindly provided by Nano Genesis Tecnologia e Inovação Ltda (Goiânia, Brazil). Niobium pentoxide (Nb_2_O_5_, ≥99.9% purity) was purchased from Sigma-Aldrich (St. Louis, MO, USA). All other reagents and solvents were of analytical grade and used as received.

### 2.2. Morphological and Chemical Characterization of Raw Powders

Before membrane incorporation, the pristine f-Gr and Nb_2_O_5_ powders were thoroughly characterized for their physicochemical properties. For morphological analysis, 1 mg of each powder was dispersed in 1 mL of deionized water, sonicated for 10 min, and 20 μL of each dispersion was drop-cast onto aluminum stubs. After drying at room temperature, samples were sputter-coated with a thin layer of Au–Pd and visualized using a Tescan MIRA3 FEG-SEM (Tescan, Brno, Czech Republic).

Functional groups were identified by Fourier-Transform Infrared Spectroscopy with Attenuated Total Reflectance (FTIR-ATR) using a Nicolet iS50 FTIR-ATR spectrometer (Thermo Fisher Scientific Inc., Waltham, MA, USA). Spectra were collected in the range of 400–4000 cm^−1^ with a resolution of 4 cm^−1^ and 64 scans. Crystal-chemistry signatures and structural information were obtained using a Thermo Scientific DXR Raman microscope (Thermo Fisher Scientific Inc., Waltham, MA, USA) equipped with a 532 nm laser. Raman data processing involved linear baseline subtraction, and deconvolution was performed using Voigt peak shapes where necessary to resolve overlapping bands.

### 2.3. Production, Purification, and Sterilization of BNC

*Gluconacetobacter hansenii* ATCC 23769 (American Type Culture Collection, Manassas, VA, USA) was revived from −80 °C glycerol stocks in Hestrin-Schramm (HS) medium. The inoculum was standardized to an optical density (OD_660_) of 0.15, diluted tenfold, and then 200 μL of this diluted inoculum was added to each well of sterile 96-well plastic plates containing 200 μL of fresh HS medium. BNC membranes were produced under static incubation conditions at 26 °C for 6 days.

The synthesized BNC membranes were purified by immersion in 0.1 M NaOH solution at 50 °C for 24 h. This was followed by extensive washing with distilled water until a neutral pH (pH 7) was achieved. Purified BNC membranes were sterilized by autoclaving at 121 °C for 20 min.

### 2.4. Post-Functionalization of BNC Membranes with Functionalized Graphite and Niobium Pentoxide

Hydrated BNC membranes were carefully drained on a sterile mesh for approximately 10 min at room temperature to remove excess water. Subsequently, 20 g (wet weight) of purified BNC were aseptically transferred to sterile 250 mL culture bottles. Separately, f-Gr and Nb_2_O_5_ were each weighed at 0.5 g and dispersed in 50 mL of deionized water using an ultrasonic bath for 10 min. to prepare individual stock suspensions.

The BNC membranes were then post-functionalized by combining them with the particulate suspensions to form four distinct experimental groups: (i) BNC: pristine BNC membrane (matrix control); (ii) BNC/f-Gr: BNC membrane combined with f-Gr suspension; (iii) BNC/Nb_2_O_5_: BNC membrane combined with Nb_2_O_5_ suspension; and (iv) BNC/f-Gr/Nb_2_O_5_: BNC membrane combined with both f-Gr and Nb_2_O_5_ suspensions. To this end, the BNC membranes and the respective particulate suspensions were combined in the culture bottles and autoclaved at 121 °C for 15 min (with a total cycle time of approximately 60 min, including heating and cooling phases) to ensure sterility and promote initial dispersion of the particulates within the BNC network. Following autoclaving, the sealed bottles were incubated at 50 °C for 7 days to facilitate in-matrix diffusion and the physical incorporation of the modifying phases. This post-synthesis route was designed to achieve a homogeneous distribution of the particulates while preserving the integrity of the cellulose nanofiber network. It is important to note that this method relies on physical adsorption and entrapment of the particles within the surface layers of the BNC network. This approach was selected to concentrate the active agents at the membrane–environment interface, where microbial contact occurs, intentionally creating a barrier layer.

### 2.5. Physicochemical Characterization of Functionalized Membranes

The micromorphology and surface topography of the functionalized membranes were examined using a Tescan MIRA3 FEG-SEM (Tescan, Brno, Czech Republic). To preserve the native nanofiber architecture for subsequent imaging, selected membrane samples were subjected to critical point drying (CPD). Membranes were gradually exchanged into anhydrous acetone and then processed in a Bal-Tec CPD 030 system (Bal-Tec AG, Balzers, Liechtenstein) using liquid CO_2_. Multiple exchanges were performed at 4–5 °C, followed by controlled heating to pass through the critical point, thereby preventing meniscus-induced collapse of the nanofiber network.

FTIR-ATR analysis (Nicolet iS50, Thermo Fisher Scientific Inc.) was performed on pristine BNC, the dual-functionalized BNC (BNC–f-Gr–Nb_2_O_5_), and the pristine f-Gr and Nb_2_O_5_ powders for comparative chemical analysis. Raman spectra were acquired using a Thermo Scientific DXR system (Thermo Fisher Scientific Inc.) with a 532 nm laser, employing 10×, 20×, 40×, and 100× objectives. The laser power was kept below 5 mW to prevent sample degradation. Spectra were collected over the range of 400–4200 cm^−1^ with linear baseline removal, and Voigt deconvolution was applied where necessary for accurate peak assignment and analysis of nanoparticle/phase components.

The crystalline structure of the carbon phase within the functionalized membranes was analyzed by X-ray diffraction. Specifically, the (002) reflection was examined to assess graphitic stacking parameters. XRD patterns were recorded using a Rigaku MiniFlex600 XRD diffractometer (Akishima-shi, Tokyo, Japan) with Cu Kα radiation (λ = 1.5418 Å) at 40 kV and 15 mA, scanning from 2~140º–0.01~100º/min.

### 2.6. Antimicrobial Assays

#### 2.6.1. Minimum Inhibitory Concentration (MIC)

The MICs of f-Gr and Nb_2_O_5_, both individually and in combination, were determined against a panel of clinically relevant microorganisms: *Candida albicans* (ATCC 10231), *Enterococcus faecalis* (ATCC 51299), *Streptococcus mutans* (ATCC UA159), and *Staphylococcus aureus* (ATCC 19095). Microorganisms were reactivated and cultured in Brain Heart Infusion (BHI; Kasvi, São José dos Pinhais, Brazil) for bacteria and Sabouraud Dextrose Broth (SDB; Kasvi, São José dos Pinhais, Brazil) for yeasts, incubated at 37 °C for 24 h. Suspensions were standardized to 0.5 McFarland (approximately 10^8^ colony-forming units per milliliter, CFU/mL, for bacteria; approximately 10^6^ CFU/mL for fungi) and subsequently diluted 1:10 to achieve final inocula of 10^7^ and 10^5^ CFU/mL, respectively.

The initial tested concentration for the powdered materials was 1 g/mL. Two-fold serial dilutions were performed in sterile 96-well round-bottom plates (*n* = 6 per dilution/condition). Each well received 100 μL of the test compound suspension (and its serial dilutions) plus 5 μL of inoculum. Positive controls contained inoculum without test compounds, while negative controls contained only culture medium and test compounds. Plates were incubated at 37 °C for 24 h under aerobic conditions. Microbial growth was assessed by visual turbidity. Wells exhibiting no visible growth were subcultured (20 μL) onto BHI or Sabouraud agar plates to confirm bactericidal/fungicidal effects. The MIC was defined as the lowest concentration of the compound at which no visible microbial growth was observed.

#### 2.6.2. Agar Diffusion Assay

The antimicrobial efficacy of the raw f-Gr and Nb_2_O_5_ powders was further assessed using the agar diffusion method against *C. albicans* (ATCC 10231) and *S. aureus* (ATCC 19433). Sterile filter paper discs (6 mm diameter, 2 mm thickness) were impregnated with 20 μL of f-Gr, Nb_2_O_5_, or their combination (at a concentration equivalent to 100 mg/mL in deionized water) and allowed to dry. These discs were then placed onto BHI agar plates previously seeded with 100 μL of microbial suspensions (3 × 10^8^ CFU/mL) after 24 h of culture. Plates were incubated at 37 °C for 24–48 h under appropriate atmospheric conditions (aerobic). Inhibition zones around the discs were measured using a digital caliper. These assays were used exclusively as preliminary screening tools for raw materials, recognizing their limitations for insoluble particulate systems.

#### 2.6.3. Modified Direct Contact Test (mDCT) for Functionalized Membranes

The antimicrobial activity of the functionalized BNC membranes was evaluated using a modified direct contact test (mDCT), a highly relevant assay for dressing performance. Membrane samples, cylindrical-shaped (6 mm diameter × 2 mm thick), were UV-sterilized for 30 min on each side. These sterilized membranes were then placed into individual wells of a 96-well plate (*n* = 6 per group). *C. albicans* and *S. aureus* (strains as listed in 2.6.1) were cultured overnight in BHI at 37 °C (aerobic conditions) and adjusted to 3 × 10^8^ CFU/mL. A 20 μL aliquot of this microbial suspension was pipetted directly onto the surface of each membrane specimen. Control wells received 20 μL of the bacterial suspension without any membrane.

After incubation at 37 °C for 1 h and 24 h, 180 μL of fresh BHI medium was added to each well and allowed to soak for 10 min to promote detachment of adhering microorganisms. From each well, 100 μL of the recovered liquid was transferred to 900 μL of sterile saline for serial dilutions. Drop-plate aliquots (3 × 20 μL) from appropriate dilutions were plated onto BHI agar and incubated for 24 h before colony-forming unit (CFU) counting. The number of viable microorganisms was determined, and the percentage reduction in CFU was calculated relative to the control wells.

### 2.7. Cytotoxicity Assay

The cytotoxic potential of the functionalized BNC membranes was assessed using extracts, in accordance with ISO 10993-12 guidelines [7], on stem cells from human exfoliated deciduous teeth (SHEDs) and human gingival fibroblasts (HGFs). SHEDs and HGFs were primary cell cultures isolated, characterized, and maintained according to protocols approved by the Institutional Ethics Committee of UFSC (CONEP B-051; Process nº. 25000.237810/2014-54).

#### 2.7.1. Extract Preparation

Membrane samples, cylindrical-shaped (6 mm diameter × 2 mm thick), were incubated in high-glucose Dulbecco’s Modified Eagle Medium (DMEM; GIBCO, Thermo Fisher Scientific Inc., Waltham, MA, USA) supplemented with 10% Fetal Bovine Serum (FBS; GIBCO, Thermo Fisher Scientific Inc., Waltham, MA, USA) for 24 h at 37 °C to obtain the extracts.

#### 2.7.2. Cell Culture and Exposure

SHEDs and HGFs (at passage 5) were seeded at a density of 2 × 10^4^ cells/well in 96-well flat-bottom plates and incubated for 24 h to allow cell attachment. The culture medium was then refreshed. Subsequently, 100 μL of each membrane extract was carefully added to the wells (*n* = 8/group), replacing the existing medium, and incubated for an additional 24 h. Untreated cells incubated with plain culture medium served as negative controls.

#### 2.7.3. Cell Viability Assessment (MTT Assay)

After the 24 h exposure, the extracts were removed, and cell viability was determined using the MTT (3-(4,5-dimethylthiazol-2-yl)-2,5-diphenyltetrazolium bromide) assay. Briefly, 50 μL of MTT solution (5 mg/mL; Sigma-Aldrich, St. Louis, MO, USA) was added per well and incubated for 3 h at 37 °C. The resulting formazan crystals were solubilized by adding 100 μL of dimethyl sulfoxide (DMSO) per well. Absorbance was measured at 540 nm with a reference wavelength of 630 nm using an Infinite M200 microplate reader (TECAN, Männedorf, Switzerland). Results were expressed as a percentage of viability relative to untreated control cells.

### 2.8. Statistical Analysis

All quantitative data were expressed as mean ± standard deviation (SD). Statistical analysis was performed using GraphPad Prism 9 (GraphPad Software, San Diego, CA, USA). The normality of residuals was assessed using Anderson-Darling, D’Agostino-Pearson omnibus, Shapiro–Wilk, and Kolmogorov–Smirnov tests, and all passed the normality assumption (*p* > 0.05). Homogeneity of variances was assessed using Brown–Forsythe and Bartlett’s tests, and no significant differences were found (*p* > 0.05). Differences between groups were analyzed using one-way ANOVA followed by Tukey’s multiple comparisons test for multiple comparisons.

## 3. Results

### 3.1. Morphological and Chemical Characterization of Raw Powders and Functionalized Membranes

**Figure 1:**
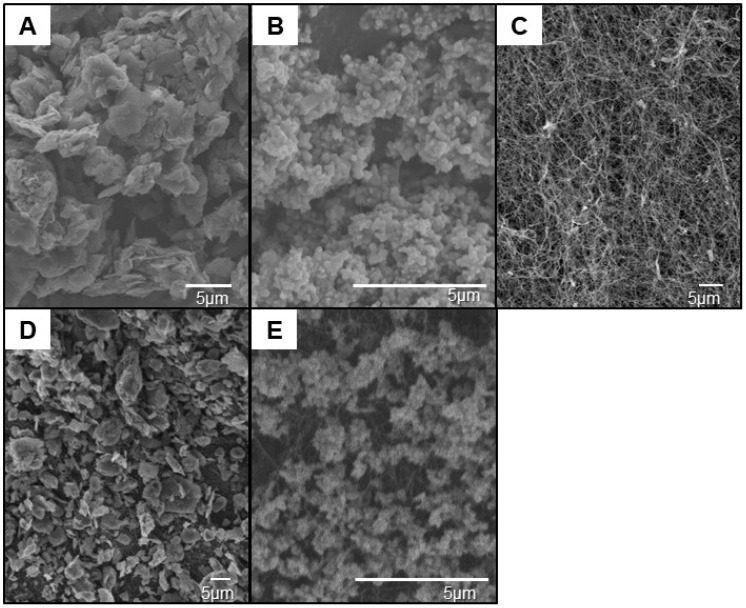
Scanning Electron Microscopy (SEM) morphological analyses of components and pristine and modified bacterial nanocellulose (BNC) membranes. (**A**) SEM micrograph of graphite particles (f-Gr) showing their characteristic morphology. (**B**) SEM micrograph of niobium pentoxide (Nb_2_O_5_) particles exhibiting their distinctive structure. (**C**) SEM micrograph of a pure nanocellulose membrane (BNC). (**D**) SEM micrograph of a nanocellulose membrane modified with graphite (BNC/f-Gr), demonstrating the effective incorporation of graphite into the matrix. (**E**) SEM micrograph of a nanocellulose membrane modified with niobium pentoxide (BNC/Nb_2_O_5_), confirming its homogeneous distribution throughout the membrane structure.

Morphological analyses of SEM micrographs, performed using ImageJ software (version 1.54r) through thresholding and particle analysis, revealed that the graphite particles exhibited an average size of 52.6 ± 14.8 μm, niobium pentoxide particles averaged 58.3 ± 6 μm, pure bacterial nanocellulose (BNC) membrane fibers had an average length of 74 ± 16.9 μm, graphite-modified BNC particles averaged 76 ± 15 μm, and niobium pentoxide-modified BNC membrane particles averaged 64.6 ± 8.9 μm (Figure 1). However, SEM analyses indicated that the graphite and Nb_2_O_5_ particles remained largely adhered to the surface of the BNC membrane, leading to partial obstruction of the native nanoporous network. This limitation reflects the constraints of the post-functionalization method used here, which promotes physical incorporation but does not ensure intimate embedding of the particles within the nanofibrous matrix. Elemental analysis was not performed in this study and represents a limitation regarding the confirmation of Nb_2_O_5_ distribution.

### 3.2. Characterization of the Functionalized Materials

#### 3.2.1. Raman Spectroscopy and Peak Deconvolution

**Figure 2:**
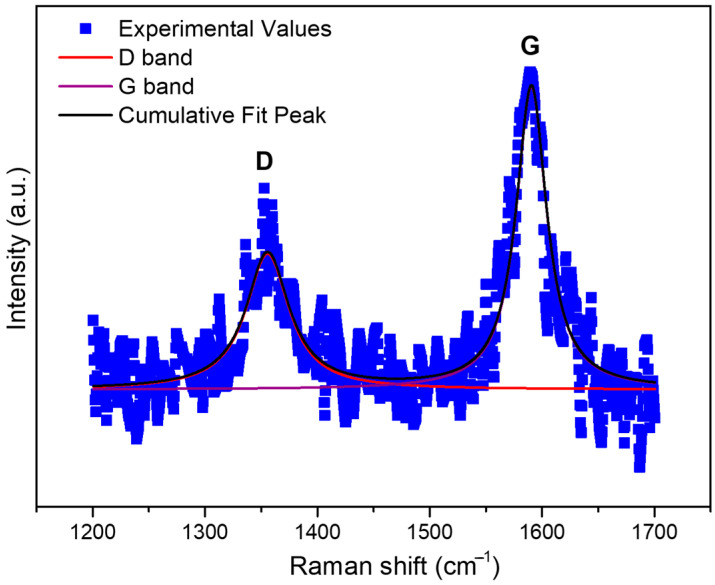
Raman spectrum of the pure functionalized graphite (f-Gr) powder used for membrane modification. The D band is centered at ~1355.7 cm^−1^ and the G band at ~1590.4 cm^−1^. The broader FWHM of the D band indicates structural disorder. The intensity ratio I_D_/I_G_ was approximately 0.64.

Raman spectra of the f-Gr material (Figure 2) displayed characteristic bands for carbonaceous materials. The D band was observed at approximately 1355 cm^−1^ and the G band at 1590 cm^−1^. Preprocessing involved a linear baseline subtraction, and the D/G region was deconvoluted using Voigt functions, resulting in a good fit (adjusted R^2^ = 0.826). The full width at half maximum (FWHM) of the D band (46.76 cm^−1^) was larger than that of the G band (33.06 cm^−1^), indicating structural disorder and the presence of edge defects, consistent with functionalized graphite (f-Gr) rather than pristine graphene. The intensity ratio I_D/I_G was determined to be approximately 0.64, further corroborating a moderate defect level within a predominantly graphitic material that retains residual functional groups [1]. This interpretation aligns with the expected identity of f-Gr and distinguishes the material from highly oxidized graphene oxides [1,2].

#### 3.2.2. X-Ray Diffraction and Layer Estimation

**Figure 3:**
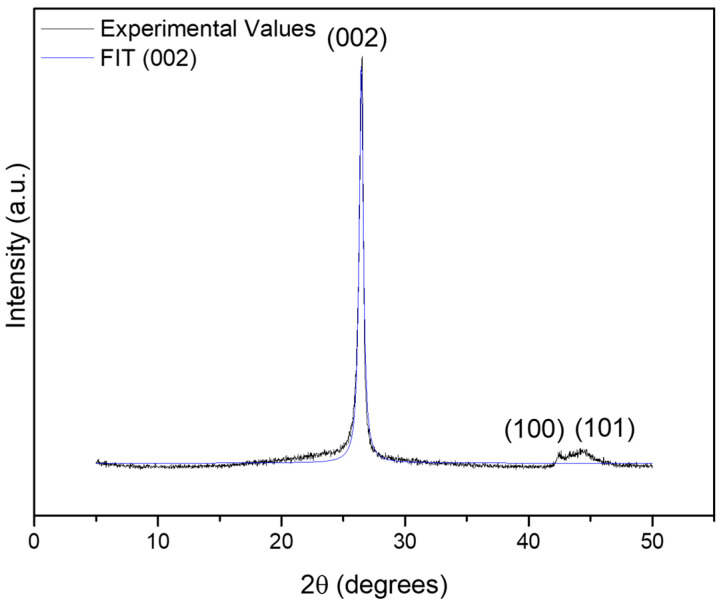
X-ray diffraction pattern of the functionalized graphite sample, highlighting the (002) reflection at 2θ ≈ 26.45°. The peak was fitted using a Voigt function, indicating high crystallinity and allowing estimation of the crystallite size and number of graphene layers.

The X-ray diffractogram of the f-Gr material (Figure 3) showed a single, well-defined peak at 2θ = 26.45°, which corresponds to the (002) plane of graphite. Voigt fitting of this peak exhibited excellent agreement with the experimental data (adjusted R^2^ = 0.98144) [1,2]. The FWHM of this peak was 0.392°. Using the Scherrer equation (with Cu Kα radiation, λ = 1.5406 Å, and shape factor K = 0.9), the crystallite thickness was estimated to be approximately 19.75 nm. Assuming an interplanar spacing of 0.335 nm, this thickness is equivalent to approximately 59 stacked graphitic layers. These results confirm that the graphitic stacking was largely preserved after functionalization, with imperfections consistent with the disorder level observed by Raman spectroscopy [1,2].

#### 3.2.3. Attenuated Total Reflectance Fourier Transform Infrared Spectroscopy (FTIR-ATR)

**Figure 4:**
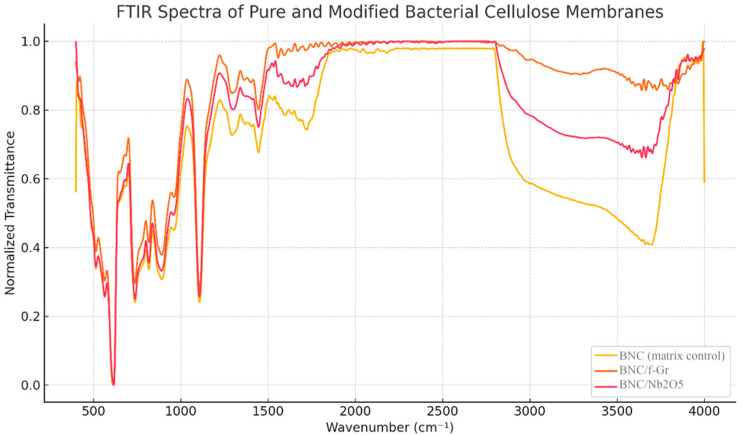
FTIR spectra of bacterial cellulose membranes: pure, with graphite, and with niobium pentoxide (Nb_2_O_5_), presented in normalized transmittance. No new absorption bands were observed following modification, but notable variations in the relative intensity of the water-associated regions (1600–3600 cm^−1^ and ~2900 cm^−1^) were recorded, reflecting differences in water content and overlap with the O–H band.

FTIR-ATR spectra of pristine BNC and BNC modified with f-Gr and/or Nb_2_O_5_ (Figure 4) exhibited the typical absorption bands associated with cellulose. These included: a broad band for O–H stretching centered around 3340 cm^−1^, the H–O–H bending vibration of adsorbed water at approximately 1630 cm^−1^, and the C–H stretching band around 2900 cm^−1^ (partially overlapped by the O–H tail). The region between 1430–1050 cm^−1^ showed characteristic bands related to skeletal and glycosidic vibrations of the cellulose backbone.

Comparison of the spectra revealed no appearance of new characteristic bands in the functionalized BNC samples, suggesting physical incorporation of the modifiers without detectable covalent bonding to the BNC backbone. A relative decrease in the intensity of water-associated regions (specifically, the broad band from 3600–1600 cm^−1^ and the C–H stretching band at ~2900 cm^−1^) was observed in the modified samples compared to pristine BNC. This decrease was more pronounced in BNC/f-Gr and BNC/f-Gr/Nb_2_O_5_ than in BNC/Nb_2_O_5_ (Figure 4), indicating spectroscopic changes associated with water-related vibrational modes. Although the dual-modified formulation was included, the specific physicochemical interactions between graphite and Nb_2_O_5_ within the BNC matrix were not deeply investigated in this study and remain a limitation to be addressed in future work.

### 3.3. Antimicrobial Activity

#### 3.3.1. Minimum Inhibitory Concentration (MIC)

The MIC values for f-Gr and Nb_2_O_5_, both individually and in combination, against a panel of microorganisms are presented in Table 1. For f-Gr: Exhibited inhibitory activity against all tested microorganisms (*C. albicans*, *E. faecalis*, *S. mutans*, and *S. aureus*) within the tested concentration range. They did not demonstrate inhibitory activity (no MIC observed) against any of the tested microorganisms up to the maximum evaluated concentration. When f-Gr and Nb_2_O_5_ were combined, no significant synergistic or antagonistic effects were observed, with MIC values remaining similar to f-Gr alone.

#### 3.3.2. Agar Diffusion Assay

Results from the agar diffusion assays against *C. albicans* and *S. aureus* (as outlined in Section 2.6.2) were consistent with the MIC findings (Table 1). Discs impregnated with f-Gr did not demonstrate clear zones of inhibition against *C. albicans* and *S. aureus*. Discs with Nb_2_O_5_ showed no measurable zones of inhibition against either microorganism. Discs containing the f-Gr/Nb_2_O_5_ combination did not produce inhibition zones for *C. albicans* and for *S. aureus*, which were similar to those observed for f-Gr alone, suggesting no synergistic effect in this assay.

#### 3.3.3. Modified Direct Contact Test (mDCT) for Functionalized Membranes

**Figure 5:**
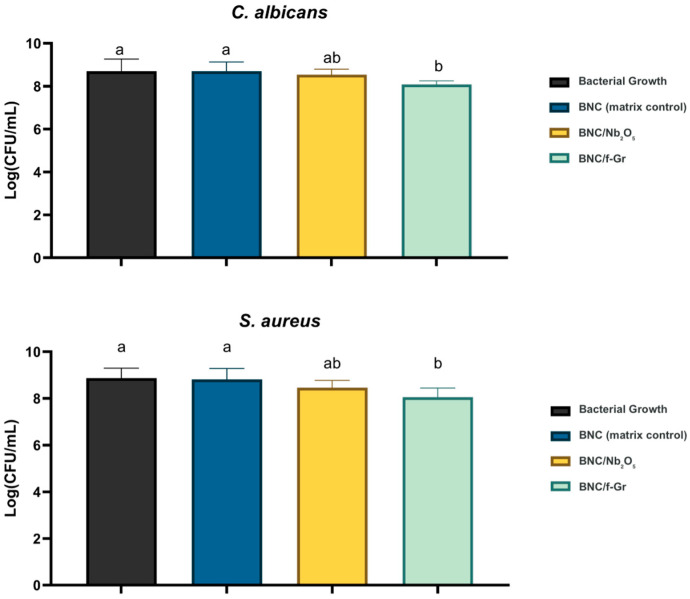
Antimicrobial activity of bacterial nanocellulose (BNC) membranes against *C. albicans* and *S. aureus* after 48 h in a direct contact test (mDCT). The graph presents bacterial growth (mean values) in the presence of pristine BNC, BNC modified with graphite (BNC/f-Gr), and BNC modified with niobium pentoxide (BNC/Nb_2_O_5_). Data are expressed as mean ± standard deviation (*n* = 6). Different lowercase letters indicate statistically significant differences among groups for the same microorganism, as determined by one-way ANOVA followed by Tukey’s post hoc test (α = 0.05). Groups sharing the same letter are not statistically different.

The modified direct contact test (mDCT) evaluated the antimicrobial activity of the functionalized BNC membranes against *S. aureus* (planktonic model, as per 2.6.3) and *C. albicans*. BNC/f-Gr membranes consistently demonstrated a significant and potent antimicrobial effect, particularly against *S. aureus*, with no detectable CFUs at both 24 h and 48 h time points. The CFU/mL counts at 48 h (Figure 5) showed distinct patterns across the groups. The BNC matrix control showed significant bacterial colonization and proliferation, with CFU/mL counts increasing from 0 to 8,7 CFU/mL at 48 h for *S. aureus* and *C. albicans*, indicating its susceptibility to microbial growth. Membranes containing only Nb_2_O_5_ behaved similarly to pristine BNC, showing no statistically significant reduction compared to BNC alone (*p* > 0.05). They did not produce a consistent and significant reduction in bacterial proliferation for *S. aureus* and *C. albicans*. Indeed, they presented an intermediate antimicrobial effect. This reduction was not statistically different from BNC/f-Gr alone. A similar trend was observed for *C. albicans*. These findings suggest no clear synergistic antimicrobial effect beyond f-Gr alone when combined with Nb_2_O_5_ in the BNC matrix under these conditions. Control wells with inoculum alone (without membranes) showed the expected microbial growth at 48 h.

These data collectively support f-Gr as the primary driver of the antimicrobial effect in the composite membranes. Nb_2_O_5_, at the loading and with the protocol used here, did not significantly enhance *S. aureus* or *C. albicans* inhibition relative to BNC alone or in combination with f-Gr.

### 3.4. Cytocompatibility (MTT)

**Figure 6:**
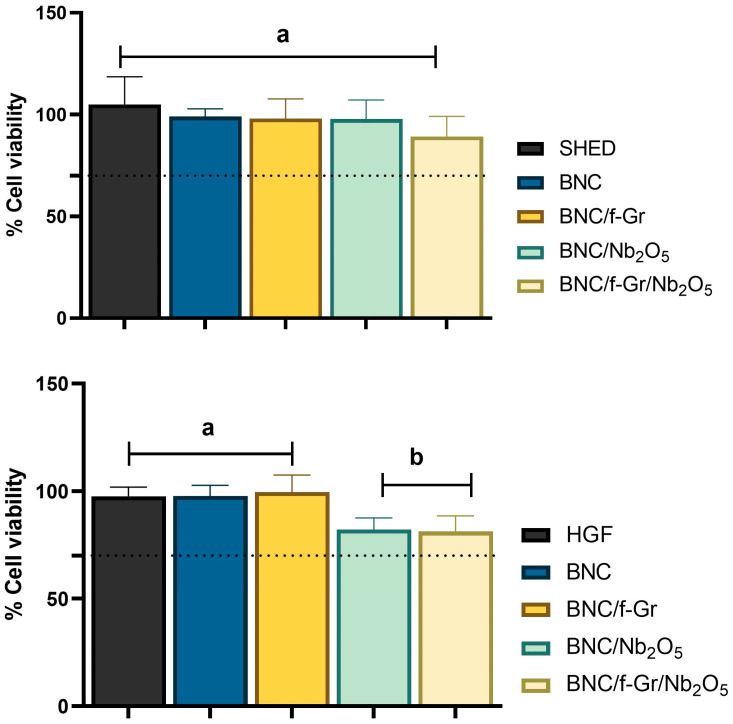
In vitro cytocompatibility of bacterial nanocellulose (BNC) membranes functionalized with functionalized graphite (f-Gr) and/or niobium pentoxide (Nb_2_O_5_), assessed by MTT assay using human gingival fibroblasts (HGFs) and stem cells from human exfoliated deciduous teeth (SHEDs). Data are presented as mean ± standard deviation (*n* = 8). Different lowercase letters indicate statistically significant differences among experimental groups within the same cell line, according to one-way ANOVA followed by Tukey’s multiple comparisons test (α = 0.05). The horizontal dashed line represents the 70% cell viability threshold established by ISO 10993-5.

Cell viability assays using extracts of the functionalized BNC membranes on SHEDs and HGFs (Figure 6) indicated that most formulations preserved cytocompatibility.

The BNC, BNC/f-Gr, and BNC/Nb_2_O_5_ groups exhibited cell viability of 99 ± 10%, 100.5 ± 14% and 105.7 ± 10%, respectively, which were comparable to the untreated cell control (100% viability) and showed no statistically significant differences (*p* > 0.05). Regarding the BNC/f-Gr/Nb_2_O_5_, this formulation showed a slight reduction in cell viability, recording 99%, 100.5% and 105.7% for SHEDs and 96%, 82,8% and 93% for HGFs. Although this reduction was statistically significant compared to the control (*p* < 0.05), it remained within the acceptability limits (≥70% viability) defined by ISO 10993-5 [7].

These results indicate that the pristine BNC matrix is highly biocompatible and that the incorporation of f-Gr and Nb_2_O_5_, individually, maintains appropriate cellular profiles under the evaluated conditions. While the combined BNC/f-Gr/Nb_2_O_5_ formulation showed a minor, statistically significant decrease in viability, it still met ISO standards for cytocompatibility [7]. It is important to note that cytocompatibility was assessed exclusively by MTT assay as an initial screening method. Additional analyses such as live/dead staining and hemolysis assays were not performed and should be included in future studies to provide a more comprehensive biological evaluation.

## 4. Discussion

Taken together, Raman/XRD/FTIR analyses confirm a graphitic carbon phase with moderate disorder (consistent with f-Gr) and physical incorporation of the modifiers into BNC [1,2,3]. FTIR analysis indicated a lack of new covalent bonds, suggesting that the retention of particles is primarily physical (entrapment and van der Waals forces). This aligns with the manufacturing method aimed at surface functionalization rather than bulk chemical modification. This structural and hydration/polarity modulation correlates with reduced bacterial proliferation, particularly evident for BNC/f-Gr, and with maintenance of cytocompatibility for the individual formulations. The f-Gr/Nb_2_O_5_ combination showed intermediate antimicrobial response and a slight viability drop, suggesting that adjustments of Nb_2_O_5_ loading and distribution may be required for additional benefits, in line with the modulatory role proposed in the literature [8,9,10,11,12].

The results show that incorporating functionalized graphite into BNC confers a consistent antimicrobial response against microorganisms relevant to chronic wounds, especially *S. aureus*, while maintaining cell viability profiles compatible with biomedical use. This performance arises from a combination of structural and surface factors: Raman and XRD signatures confirm a predominantly graphitic phase with moderate disorder, whereas FTIR-ATR indicates physical incorporation of the modifiers and a relative reduction of water-associated bands, more evident in the presence of functionalized graphite. Direct measurements of surface wettability, such as water contact angle analysis, were not performed and therefore definitive conclusions regarding surface polarity cannot be drawn. These findings point to modulation of BNC hydration state and surface polarity, features that influence protein adsorption, cell–material interactions, and particularly microbial adhesion and proliferation [13,14].

Regarding antimicrobial activity, the results indicated a bacteriostatic rather than a strongly bactericidal effect. The absence of inhibition zones and defined MIC values reinforces that the antimicrobial behavior of the materials is contact-dependent, which is more appropriately assessed by the modified direct contact test. As highlighted by the microbiological assays, f-Gr was the primary driver of this effect. The lack of a ‘kill-zone’ in diffusion assays suggests that the mechanism is contact-dependent, requiring direct interaction between the bacteria and the modified surface, rather than the release of active ions. While the reduction in CFU was not total, the significant suppression of *S. aureus* proliferation compared to the control validates the potential of f-Gr as a surface modulator to control bioburden. SEM analysis revealed that f-Gr and Nb_2_O_5_ particles were predominantly located on the BNC surface, partially occluding the native porosity, for a barrier dressing application, this surface coverage can be advantageous [15,16]. It creates a physical shield that may prevent bacterial penetration into the wound bed, although it may also impact gas exchange, a factor that requires optimization in future studies [3,4]. This balance is particularly pertinent in chronic wounds, where high microbial burden and persistent inflammation hinder healing progression [17,18].

Carbon-based materials, including graphitic structures, have been reported to exert antimicrobial effects primarily through contact-dependent mechanisms. These include physical disruption of bacterial membranes, induction of oxidative stress at the cell–material interface, and interference with bacterial adhesion and biofilm formation. The absence of inhibition halos in diffusion assays and the significant effects observed in direct contact tests support a surface-mediated mechanism in the present study.

For niobium pentoxide, the data indicate behavior similar to pristine BNC under the evaluated conditions, which is consistent with its function as an inorganic modulator of surface and stability reported in composites and ceramic systems [19]. The combined functionalized graphite plus Nb_2_O_5_ formulation showed an intermediate antimicrobial response, accompanied by a slight reduction in cell viability that remained within acceptable parameters, indicating that under the present conditions, the dominant contribution to bacterial inhibition arises from the carbonaceous component. The convergence of graphitic identity confirmed by Raman and XRD, hydration and polarity modulation evidenced by FTIR-ATR, and the microbiological and cytocompatible performance observed in the assays, reinforces the coherence of the proposed materials design with the functional requirements of dressings for chronic wounds [20].

We acknowledge that chronic wounds involve complex pathophysiology beyond bacterial infection, including oxidative stress and persistent inflammation. While this study focused specifically on the antimicrobial and cytocompatibility aspects, the potential antioxidant properties of carbon-based materials and niobium species—not tested here—remain a subject for future investigation.

When the biological performance of the membranes is compared with that of the raw materials, it becomes evident that functionalized graphite is the dominant contributor to the observed antimicrobial effect. Raw Nb_2_O_5_ powders showed no detectable inhibitory activity in screening assays, behaving similarly to pristine BNC when incorporated into the membrane. These findings indicate that, at the loading and dispersion achieved in this study, Nb_2_O_5_ does not play a decisive antimicrobial role, but rather acts as a secondary inorganic modifier.

## 5. Conclusions

This study demonstrated that the surface post-functionalization of BNC with f-Gr results in a composite capable of limiting bacterial proliferation through a moderate, contact-dependent mechanism, particularly against *S. aureus*, while maintaining acceptable cytocompatibility. The incorporation of Nb_2_O_5_ alone did not significantly enhance antimicrobial activity under the tested conditions. While the method results in pore occlusion and physical adhesion of particles, this surface modification strategy successfully alters the polarity and hydration profile of BNC. These findings suggest that BNC/f-Gr composites have potential as barrier membrane with potential application in wound management to limit bacterial proliferation, although further optimization is required to address mechanical properties and evaluate efficacy in complex wound environments.

Nb_2_O_5_ behaved as an inorganic additive that did not significantly alter antimicrobial performance under the experimental conditions evaluated in this study, showing behavior similar to pristine BNC when used alone and an intermediate effect when combined with functionalized graphite. Overall, the BNC formulation with functionalized graphite emerges as the most promising among those evaluated for dressings aimed at the management of chronic wounds, reconciling microbial control and cytocompatibility within parameters required for biomaterials of clinical interest. Future work will focus on optimizing modifier loading and distribution, and exploring additional properties such as mechanics, conductivity, and performance in biofilm models.

## Figures and Tables

**Table 1:** Minimum inhibitory concentration (MIC) and agar diffusion results for pristine bacterial nanocellulose (BNC) and BNC membranes functionalized with functionalized graphite (f-Gr) and/or niobium pentoxide (Nb_2_O_5_).

Groups	MIC	Agar Diffusion (mm)
BNC (matrix control)	__	6 ± 1.2
BNC/Nb_2_O_5_	__	6 ± 1.8
BNC/f-Gr	__	6 ± 2.1
BNC/f-Gr/Nb_2_O_5_	__	6 ± 1.7

__ means that no MIC was detected.

## Data Availability

The original contributions presented in the study are included in the article; further inquiries can be directed to the corresponding author.

## Abbreviations

The following abbreviations are used in this manuscript:

BNC	Bacterial nanocellulose (pristine BNC membrane, matrix control)
BNC/f-Gr	Bacterial nanocellulose membrane combined with functionalized graphite suspension
BNC/Nb_2_O_5_	Bacterial nanocellulose membrane combined with niobium pentoxide suspension
BNC/f-Gr/Nb_2_O_5_	Bacterial nanocellulose membrane combined with both functionalized graphite and niobium pentoxide suspensions
BHI	Brain Heart Infusion
CFU/mL	Colony-forming units per milliliter
DMEM	Dulbecco’s Modified Eagle Medium
DMSO	Dimethyl sulfoxide
ECM	Extracellular matrix
FBS	Fetal Bovine Serum
f-Gr	Functionalized grafite
FTIR-ATR	Fourier-Transform Infrared Spectroscopy with Attenuated Total Reflectance
FWHM	Full width at half maximum
HGFs	Human Gingival Fibroblasts
HS medium	Hestrin-Schramm médium
ISO	International Organization for Standardization
mDCT	Modified Direct Contact Test
MIC	Minimum Inhibitory Concentration
MTT	(3-(4,5-dimethylthiazol-2-yl)-2,5-diphenyltetrazolium bromide)
Nb_2_O_5_	Niobium Pentoxide
OD660	Optical density at 660 nm
SDB	Sabouraud Dextrose Broth
SD	Standard deviation
SEM	Scanning Electron Microscopy
SHEDs	Stem Cells from Human Exfoliated Deciduous Teeth
XRD	X-ray diffraction
